# Enhanced methane emissions from tropical wetlands during the 2011 La Niña

**DOI:** 10.1038/srep45759

**Published:** 2017-04-10

**Authors:** Sudhanshu Pandey, Sander Houweling, Maarten Krol, Ilse Aben, Guillaume Monteil, Narcisa Nechita-Banda, Edward J. Dlugokencky, Rob Detmers, Otto Hasekamp, Xiyan Xu, William J. Riley, Benjamin Poulter, Zhen Zhang, Kyle C. McDonald, James W. C. White, Philippe Bousquet, Thomas Röckmann

**Affiliations:** 1 Institute of Marine and Atmospheric Research Utrecht (IMAU), Utrecht, The Netherlands; 2 SRON Netherlands institute for Space Research, Utrecht, The Netherlands; 3 Department of Meteorology and Air Quality (MAQ), Wageningen University and Research Centre, WageningenThe Netherlands.; 4 Department of Physical Geography and Ecosystem Science, Lund University, Lund, Sweden; 5 NOAA Earth System Research Laboratory, Boulder, Colorado, USA; 6 Earth Sciences Division, Lawrence Berkeley National Laboratory, Berkeley, California, USA; 7 CAS Key Laboratory of Regional Climate-Environment for Temperate East Asia, Institute of Atmospheric Physics, Beijing, China; 8 Institute on Ecosystems and Department of Ecology, Montana State University, Bozeman, USA; 9 Swiss Federal Research Institute WSL, Birmensdorf, Switzerland; 10 City College of New York, City University of New York, New York, NY, USA; 11 Institute of Arctic and Alpine Research, Boulder, CO, USA; 12 Laboratoire des Sciences du Climatet de l’Environnement (LSCE), Gif-sur-Yvette, France

## Abstract

Year-to-year variations in the atmospheric methane (CH_4_) growth rate show significant correlation with climatic drivers. The second half of 2010 and the first half of 2011 experienced the strongest La Niña since the early 1980s, when global surface networks started monitoring atmospheric CH_4_ mole fractions. We use these surface measurements, retrievals of column-averaged CH_4_ mole fractions from GOSAT, new wetland inundation estimates, and atmospheric *δ*^13^C-CH_4_ measurements to estimate the impact of this strong La Niña on the global atmospheric CH_4_ budget. By performing atmospheric inversions, we find evidence of an increase in tropical CH_4_ emissions of ∼6–9 TgCH_4_ yr^−1^ during this event. Stable isotope data suggest that biogenic sources are the cause of this emission increase. We find a simultaneous expansion of wetland area, driven by the excess precipitation over the Tropical continents during the La Niña. Two process-based wetland models predict increases in wetland area consistent with observationally-constrained values, but substantially smaller per-area CH_4_ emissions, highlighting the need for improvements in such models. Overall, tropical wetland emissions during the strong La Niña were at least by 5% larger than the long-term mean.

CH_4_ is the second most important anthropogenic greenhouse gas after CO_2_, accounting for 20% of direct anthropogenic radiative forcing^1^. CH_4_ contributes strongly to anthropogenic climate change, directly through its radiative forcing as well as indirectly through impacts on atmospheric chemistry^2^. With a relatively short atmospheric lifetime of ∼9 years, CH_4_ is a primary target for global warming mitigation strategies^3^. Over the past decades, the atmospheric CH_4_ growth rate has been highly variable^3^,^4^,^5^,^6^,^7^, including an approximate stabilization from 1999 to 2006 followed by a renewed growth since 2007^8^. Among a range of explanations that were proposed, some studies have suggested that more than 70% of the interannual variations of CH_4_ can be explained by wetland CH_4_ emissions^9^,^10^.

Wetland CH_4_ emissions are highly sensitive to soil temperature and moisture^11^. Paleo records and studies of contemporary CH_4_ suggest a strong positive feedback of wetlands to global warming through CH_4_ emissions^12^,^13^. Proper quantification of this feedback is important for accurate future climate projections. Therefore, it is crucial to better understand the sensitivity of wetland CH_4_ emissions to changes in climatic parameters. The El Niño Southern Oscillation (ENSO) is a major mode of variability of global precipitation and temperature, comprising alternating El Niño and La Niña phases^14^. Hodson *et al*.^15^ estimated the influence of precipitation and temperature change, driven by ENSO, on wetland CH_4_ emissions using a process-based wetland model. They found that a large fraction of CH_4_ variability is correlated with ENSO, with higher tropical wetland CH_4_ emission during La Niña periods. However, this pattern has not been verified until now by atmospheric CH_4_ measurements during a La Niña. Furthermore, La Niña periods have received less attention in studies of the atmospheric CH_4_ budget than El Niño, since continued warming likely favors neutral or El Niño conditions^16^.

The La Niño of 2011 (LN11 hereafter) was the strongest since 1980 (see Fig. 1a) and offers the possibility to investigate the response of the atmospheric CH_4_ budget to La Niña conditions. In this study, we investigate this response by combining different measurement dataset and model simulations. A brief overview of them is given in the next section.

## Method and Data

Atmospheric CH_4_ measurements are available during the 2011 La Niña period from ground-based networks (NOAA-ESRL, CSIRO), and space (GOSAT, SCIAMACHY). The Greenhouse Gases Observing Satellite (GOSAT) has been measuring spectra for retrieval of the column average mole fraction of CH_4_ (XCH_4_) since June 2009^17^. Onboard GOSAT is the Thermal And Near infrared Sensor for carbon Observation-Fourier Transform Spectrometer (TANSO-FTS), from which XCH_4_ is obtained with high sensitivity to the lower troposphere, and hence, to surface emissions^18^. We analyze the interannual variability in GOSAT full-physics (FP) XCH_4_, obtained using the RemoteC algorithm^19^, and ground-based CH_4_ flask-air measurements. Supplementary Material (SM) Section 9 further explains the FP retrieval method and justifies our choice of FP XCH_4_ over XCH_4_ derived from other retrieval algorithms.

In addition to the surface emissions, changes in atmospheric transport can cause interannual variability in CH_4_^20^. Large-scale transport patterns, including the strength of inter-hemispheric exchange and atmospheric temperature are influenced by ENSO^3^,^21^,^22^,^23^. To quantify the contribution of these meteorological parameters, we ran the Tracer Transport Model version 5 (TM5^24^) repeating surface emissions of 2008 for every year in 2009–2015. This simulation is referred to as TM5-Meteorology from hereon (see SM Section 1). To quantify the contribution of the surface emissions to XCH_4_ variability, we look at the difference between GOSAT FP XCH_4_ and XCH_4_ sampled from TM5-Meteorology.

Atmospheric inverse modeling systems are well established tools to convert atmospheric CH_4_ measurements into surface emissions^25^,^26^. We use the TM5-4DVAR (TM5-variational data assimilation system^27^) in combination with GOSAT FP XCH_4_, and surface measurements from NOAA-ESRL^3^ and CSIRO^28^ to optimize surface CH_4_ emissions. Note that the inverse model makes use of actual meteorological fields from the ECMWF ERA-interim reanalysis to account for variability in the atmospheric transport of CH_4_. Earlier studies have established the link between biomass burning CH_4_ emissions and ENSO^29^. To exclude the influence of biomass burning, fire related CH_4_ emissions from the Global Fire Emissions Database version 4s (GFED4s) inventory have been subtracted from the TM5-4DVAR emissions.

The origin of an atmospheric CH_4_ anomaly can be identified using CH_4_ stable isotope measurements. We look at measurements of ^13^C/^12^C in CH_4_ (expressed in *δ*-notation as *δ*^13^C-CH_4_) analyzed by INSTAAR in samples from the NOAA-ESRL (ref. 30, see Fig. 2b). *δ*^13^C-CH_4_ of atmospheric CH_4_ (global average in 2009=−47.14‰) is controlled by the relative contribution from different source types with distinct isotopic signatures. The mean isotopic signatures of the biogenic category is ∼ −60‰ (includes wetlands, agriculture, waste), for the thermogenic category it is ∼ −37‰ (includes fossil-fuels) and for pyrogenic category it is ∼ −22‰ (includes biomass burning)^31^. To account for impact of meteorological variability on *δ*^13^C-CH_4_, a meteorology simulation of *δ*^13^C-CH_4_ was performed using TM5^32^.

To identify factors which might have altered the wetland emissions, we look at the variability in land precipitation and temperature data in CRU-TS version 3.23 (Climatic Research Unit-time series^33^). We also analyze CH_4_ emission and surface inundation extent from two process-based wetland models: LPJ-wsl^15^,^34^ and CLM4.5^35^,^36^. Additionally, we derive an independent estimate of inundation extent from remotely sensed Surface WAter Microwave Product Series (SWAMPs^37^).

The primary sink of CH_4_ is the reaction with OH in the troposphere (∼454–617 TgCH_4_ yr^−1^ ^2^), and interannual variations in OH can also contribute to the observed CH_4_ variability. Tropospheric OH concentrations are influenced by many factors, including temperature, water vapor, O_3_, NO_x_, CH_4_, CO, and the overhead stratospheric ozone column^38^. The TM5-4DVAR inversions performed in this study make use of OH fields from ref. 39, which vary seasonally, but are the same each year. To investigate possible variations caused by the OH sink, we analyze posterior CH_4_ emissions of LMDz-PYVAR-SACS inversion^40^,^41^,^42^. In this inversion, the OH fields were optimized simultaneously using methyl chloroform (MCF) measurements (see SM Section 1.3).

### Data Analysis

The above mentioned measurements and model outputs have been analyzed by taking their monthly averages and integrating them over three zones over the globe (GLO) : Tropics (TRO: 30°S to 30°N), Northern Extra Tropics (NET: 30°N to 90°N), Southern Extra Tropics (SET: 90°S to 30°S). The time series of these monthly averages have been detrended and smoothened using a 12 month running mean. The resulting time series of GOSAT FP XCH_4_ from this method is less influenced by systematic errors, which often affect the satellite retrievals^9^,^43^,^44^.

**Figure 1:**
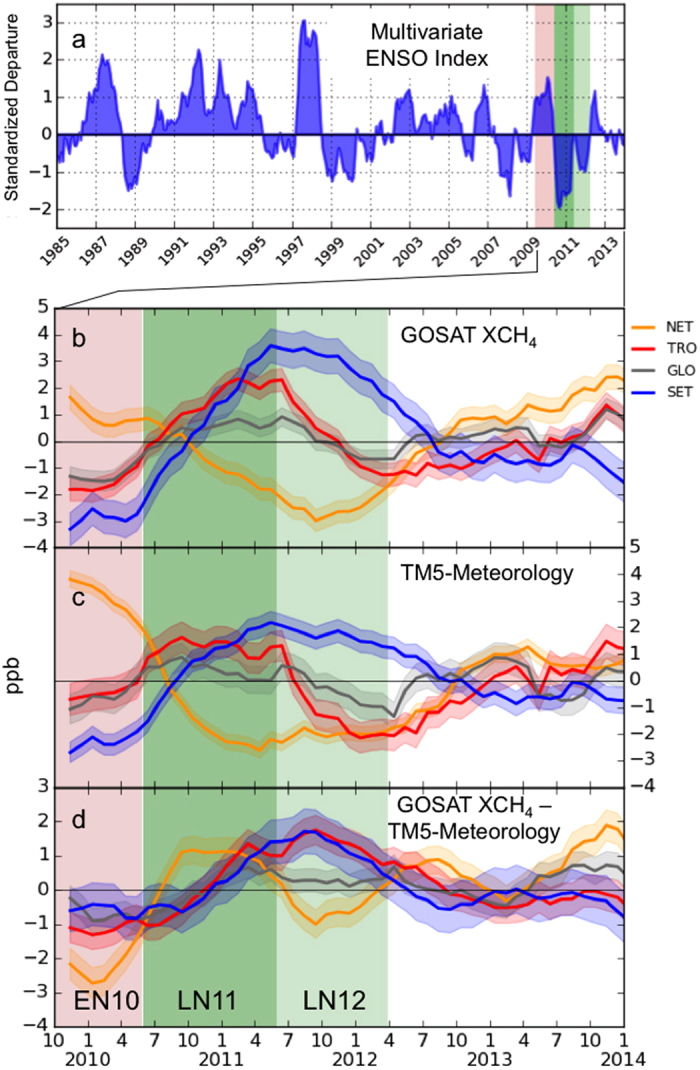
(**a**) Multivariate ENSO index (MEI^53^). The strong La Niña of 2011 (LN11) is shaded in dark green. The preceding El Niño of 2010 (EN10) and succeeding weak La Niña of 2012 (LN12) are shaded in lighter red and green colors, respectively. (**b,c,d**) Detrended and smoothened XCH_4_ integrated over the large regions: (**b**) GOSAT FP XCH_4_, (**c**) TM5-Meteorology XCH_4_—that is, XCH_4_ variability due to meteorological changes (TM5 is run with annually repeating emissions). (**d**) GOSAT FP XCH_4_ corrected for the influence of meteorology (the difference between b and c). The light shaded regions represent the ±1*σ* uncertainty of the respective time series. NET, SET, TRO, and GLO are abbreviation of Northern Extra Tropics, Southern Extra Tropics, Tropics and Globe, respectively.

Further, based on the Multivariate ENSO index (MEI) we define three periods: 1) La Niña in 2011 as LN11; 2) The preceding El Niño of 2010 as EN10; 3) succeeding weak La Niña of 2012 as LN12 (see Fig. 1a). Time series of measurements and model outputs are analyzed during these periods using the following parameters:

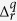
: Defined as the change of a quantity *q* in region *r* during an ENSO phase.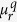: Defined as the mean of a quantity *q* in region *r* during an ENSO phase.

**Figure 2:**
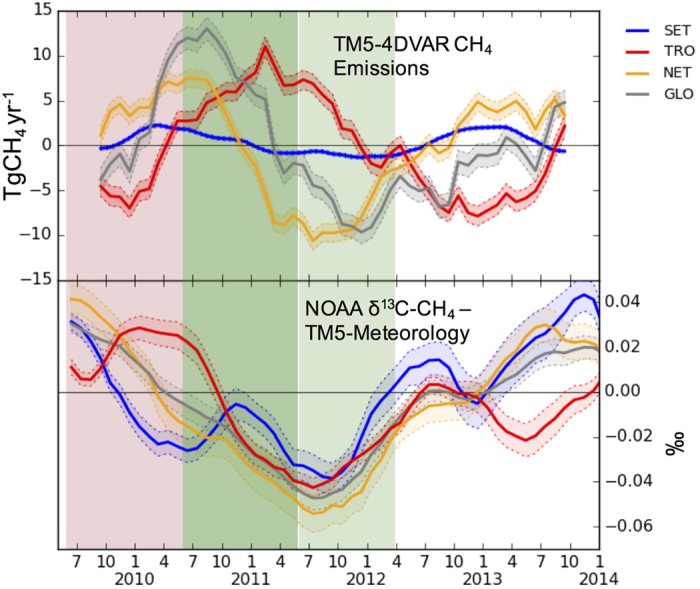
(**a**) Detrended and smoothened CH_4_ surface emissions estimates from TM5-4DVAR for the same regions as in Fig. 1. The variability of GFED4s biomass burning emissions has been subtracted. (**b**) *δ*^13^C-CH_4_ measurements^54^ corrected for the influence of transport using a meteorology-only TM5 simulation of *δ*^13^C-CH_4_^32^. The light shaded regions represent the ±1*σ* uncertainty of the respective time series.

Table 1 summarizes 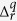 or 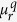 of time series shown in Figs 1 and 2.

## Results and Disscussion

### GOSAT observations

Figure 1b shows detrended and smoothened time series of GOSAT FP XCH_4_. During LN11, SET and TRO have increasing XCH_4_ (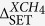 = 5.6ppb and 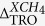 = 2.4) contrasted by a decrease in NET (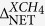 = −2.7 ppb). During LN12, the opposite trends are found: XCH_4_ gradually reduced in SET and TRO and increased in NET.

Figure 1c shows the results of TM5-Meteorology simulation. The modeled XCH_4_ decreases over NET and increases over SET (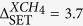 ppb, 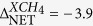 ppb). These trends can be attributed to the faster inter-hemispheric exchange during La Niña conditions, which transferred additional CH_4_ rich air from the Northern Hemisphere to the Southern Hemisphere. Francey *et al*.^21^ found the strongest inter-hemispheric transport of CH_4_ during LN11 since 1990. Previous studies have reported such enhancement in inter-hemispheric transport, and consequent increase in CH_4_ in the Southern Hemisphere, during the La Niña of 2007–2008^3^ and 1989^23^. During LN12, the strength of anomalies over both regions weakens as inter-hemispheric exchange returns to its normal strength. Modeled XCH_4_ over TRO show less variation during LN11. During LN12, the modeled XCH_4_ (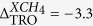 ppb) explains a major fraction of GOSAT XCH_4_ (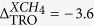 ppb) variability. It is noteworthy that OH concentrations are not affected in our meteorology simulation as TM5 uses OH fields from Spivakovsky *et al*.^39^. Eventhough these fields don’t vary interannually, temperature variations can still cause variability in the atmospheric CH_4_ sink, as the rate constant for reaction with OH is temperature dependent^45^.

Figure 1d shows the GOSAT FP XCH_4_ after correction for variations in atmospheric transport using the TM5-Meteorology simulation, and indicates the fraction of CH_4_ variability that can be attributed to variability in CH_4_ sources and sinks. An analogous plot showing surface flask-air measurements is given in the SM section 4 and shows similar patterns. During LN11, the largest transport-corrected 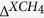 is seen in TRO (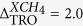 ppb), pointing to higher CH_4_ emissions from the tropical continents. As SET does not have large CH_4_ surface emissions, it is caused most likely by transport from TRO. This means the transport-corrected anomaly of TRO is transferred to SET.

### Emissions and source attribution

Detrended and smoothened time series of the posterior TM5-4DVAR emissions are shown in Fig. 2a. The CH_4_ emissions over TRO show a positive anomaly during LN11 (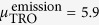 TgCH_4_ yr^−1^). 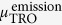 is higher by 11.7 TgCH_4_ yr^−1^ in LN11 than in the preceding EN10 (
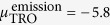
 TgCH_4_ yr^−1^). This can be due to higher CH_4_ emissions from tropical wetlands during the La Niña, as suggested by ref. 15. During LN12, the CH_4_ emission enhancement over TRO is weaker (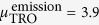 TgCH_4_ yr^−1^). NET has a sharp decrease in CH_4_ emissions during LN11 (
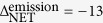
 TgCH_4_ yr^−1^), which shifts the maximum of the global CH_4_ emission anomaly towards the beginning of LN11.

Detrended and smoothened time series of *δ*^13^C-CH_4_ is shown in Fig. 2b. During LN11, *δ*^13^C-CH_4_ decreased over each region. Over TRO, we observe a decrease of 0.06‰, which is a larger decrease than the GLO *δ*^13^C-CH_4_ decrease by 0.03‰. An isotope mass balance calculation shows that if the increase in TRO CH_4_ emissions of 11.7 TgCH_4_ yr^−1^ (change from EN10 with 
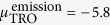
 TgCH_4_ yr^−1^ to LN11 with 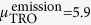 TgCH_4_ yr^−1^), is attributed to a biogenic source, it would cause a drop in *δ*^13^C-CH_4_ of similar magnitude. This indicates that the source of the LN11 CH_4_ anomaly in the Tropics is of biogenic origin. The increase of GLO *δ*^13^C-CH_4_ (≈0.03‰) over LN12 can be explained by reduced biogenic emissions (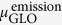
(LN12) - 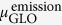
(LN11) = ∼ −14 TgCH_4_ yr^−1^) and increased biomass burning emissions (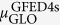 (LN12) – 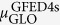 (LN11) = 2.2 TgCH_4_ yr^−1^) in comparison to LN11.

Tropical Biomass burning is strongly influenced by ENSO^46^. Globally, GFED4s biomass burning CH_4_ emissions indicate a decrease of 0.67 TgCH_4_ yr^−1^ from EN10 to LN11 (see SM Section 5). The effect of this change on the isotopic composition is only −0.003‰, thus much smaller than the observed trend. According to GFED4s, CH_4_ emissions from biomass burning over TRO during LN11 are close to average over the whole period (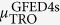 = −0.23 TgCH_4_ yr^−1^). In LN12, these emissions were higher in NET and TRO (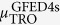 = 1.09 TgCH_4_ yr^−1^ and 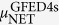 = 1.32 TgCH_4_ yr^−1^). This increase may be explained by higher fuel availability due to enhanced biomass growth during the preceding LN11. Ref. 47 suggested a similar impact of Australian biomass burning on CO_2_ emissions.

**Figure 3:**
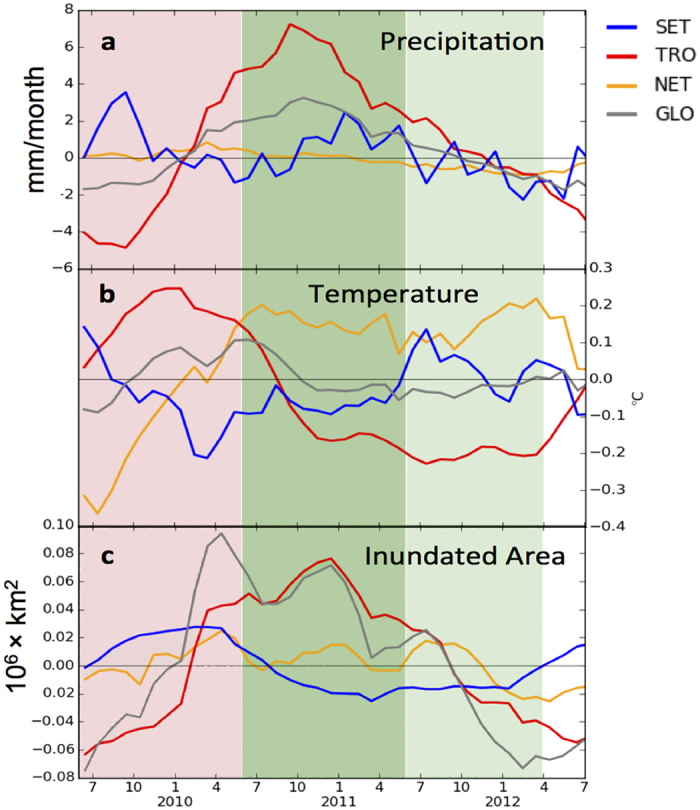
(**a,b**) Detrended and smoothened regionally averaged precipitation and temperature measurements over land in CRU-TS version 3.23 (Climatic Research Unit-time series^33^). (**c**) Anomalies in the total inundated area estimated by SWAMPS^37^.

Figure 3a and b show monthly anomalies recorded in climate parameters. A significant redistribution of heat and precipitation is seen during the different phases of ENSO. 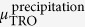 was −1.72, 4.90, and 0.64 mm during EN10, LN11, and LN12, respectively. During LN11 the precipitation anomaly in TRO (and in GLO) was the highest since the onset of the 21st century (see SM Figure 10). Regions like Australia had six consecutive seasons of increased rainfall over the La Niña of 2011 and 2012^48^. Higher temperatures were observed in NET during LN11 (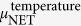 = 0.18 °C), favoring increased biomass burning, for example, near Moscow during the summer of 2010^49^,^50^. Mean temperatures during LN11 (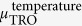 = −0.05 °C) were in between those during EN10 (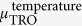 = 0.15 °C) and LN12 (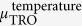 = −0.22 °C).

An increase in total inundated area is observed in the remotely sensed SWAMPS data (see Fig. 3c). The total inundated area estimated by the wetland models LPJ-wsl and CLM4.5 also show a similar increase. However, these wetland models estimate a relatively weaker enhancement in CH_4_ emissions with 
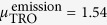
 TgCH_4_ yr^−1^ for LPJ-wsl and 
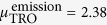
 TgCH_4_ yr^−1^ for CLM4.5 during LN11 (see SM Section 8).

To further investigate the relation between inversion-estimated CH_4_ emissions and potential climatic drivers, we examine their correlation coefficients (R) [see SM Figure 11]. CH_4_ emission anomalies (as shown in Fig. 2a) correlate stronger with precipitation anomalies than with temperature anomalies (as shown in Fig. 3) in both NET 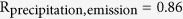, 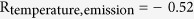) and TRO (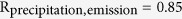, 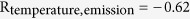). This points to precipitation as the more important driver of the CH_4_ anomaly in TRO during LN11, supported further by the correlation with inundated area (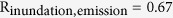). This is consistent with the findings of Bloom *et al*.^51^, who show that precipitation plays a more dominant role than temperature in determining anomalous CH_4_ variability in the Tropics.

To investigate possible variations caused by the OH sink, we analyzed optimized CH_4_ emissions from a LMDz-PYVAR-SACS inversion, in which OH fields were also optimized. During LN11, the results of this inversion suggest 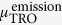 of 9.1 TgCH_4_ yr^−1^, compared to TM5-4DVAR 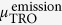 of ∼6 TgCH_4_ yr^−1^ (see SM Section 6). The differences in interannual variations of the emission estimates of the two inversions are mainly caused by their different treatment of OH sink. Assuming that the MCF-optimized OH sink of LMDz-PYVAR-SACS is more accurate than TM5, the sink was stronger than normal during LN11 by ∼3 TgCH_4_ yr^−1^. This is consistent with the hypothesis of an increased CH_4_ sink during La Niña and a weaker sink during El Niño^52^.

## Conclusion

Our inversion results, supported by *δ*^13^C-CH_4_ measurements, provide strong evidence of enhanced tropical biogenic CH_4_ emissions by ∼6–9 TgCH_4_ yr^−1^, during the La Niña of 2011. Wetlands were the likely cause of this anomaly as a simultaneous increase in total inundated area is shown by remote sensing observations and hydrological models. The increase in inundated area was in response to La Niña induced increase in precipitation. 2011 experienced the strongest La Niña event in the past 4 decades as well as since the onset of modern atmospheric CH_4_ measurements. It is noteworthy that during this La Niña the increase in global CH_4_ mole fractions were not as pronounced due to a simultaneous decrease in the CH_4_ emissions in the Northern Extra Tropics. Our analysis presents the first evidence of the large-scale response of wetland CH_4_ emissions to ENSO variability using satellite retrievals.

## Data Availability

We use Level 2 SRFP XCH4 v2.3.7 GOSAT XCH4 retrievals that are publicly available from ESA’s Climate Change Initiative website (www.esa-ghg-cci.org/). NOAA CH_4_ and INSTAAR δ13C-CH_4_ measurements are freely available from NOAA’s public ftp server (ftp://aftp.cmdl.noaa.gov/data). CSIRO CH_4_ measurements can be downloaded from the WDCGG (World Data Centre for Green-house Gases) website. GFED4s CH_4_ emissions can be downloaded from http://daac.ornl.gov. CRU TS3.23). Precipitation and temperature data are held at British Atmospheric Data Centre, RAL, UK (http://badc.nerc.ac.uk/data/cru/). SWAMP wetlands fraction data can be downloaded from http://wetlands.jpl.nasa.gov after a short registration. CLM4.5 and LPJ-wsl CH_4_ emissions and wetlands fractions can be obtained by contacting William J. Riley and B. Poulter, respectively.

## Additional Information

**How to cite this article:** Pandey, S. *et al*. Enhanced methane emissions from tropical wetlands during the 2011 La Niña. *Sci. Rep.* **7**, 45759; doi: 10.1038/srep45759 (2017).

**Publisher's note:** Springer Nature remains neutral with regard to jurisdictional claims in published maps and institutional affiliations.

## Supplementary Material

Supplementary Information

## Figures and Tables

**Table 1:** 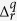
 (i.e. the sum of the derivative) or 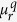 (i.e. mean) of the times series of quantity *q*, averaged over region *r*, as shown in Figs 1 and 2 during different ENSO phases.

Region	Phase	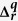			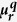	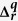
		GOSAT (ppb)	TM5 (ppb)	GOSAT-TM5 (ppb)	TM5-4DVAR (TgCH_4_ yr^−1^)	*δ*^13^C-CH_4_ (‰)
NET:	EN10	−0.85	−1.60	0.74	4.58	−0.05
	LA11	−2.66	−3.86	1.20	0.01	−0.03
	LA12	0.31	0.38	−0.08	−8.91	0.03
TRO:	EN10	0.86	0.61	0.24	−5.76	0.01
	LA11	2.42	0.38	2.04	5.94	−0.06
	LA12	−3.57	−3.32	−0.25	3.94	0.02
SET:	EN10	0.55	0.77	−0.23	0.21	−0.06
	LA11	5.62	3.70	1.92	0.63	−0.01
	LA12	−1.73	−0.78	−0.94	−1.14	0.03
GLO:	EN10	0.56	1.18	−0.61	−0.96	−0.03
	LA11	0.91	−0.50	1.41	7.58	−0.03
	LA12	−1.59	−1.54	0.05	−6.11	0.03

Please note that the GOSAT and TM5-4DVAR time series do not cover the whole EN10 period, as continuous GOSAT measurements are only available since June 2009, and the 12-month smoothing causes data points loss. Only *δ*^13^C-CH_4_ values cover the whole EN10.
