# Supplementary material for: Enhanced methane emissions from tropical wetlands during the 2011 La Niña
Source: Sci Rep. 2017 Apr 10;7:45759. doi: 10.1038/srep45759 (PMC5385533; doi:10.1038/srep45759)
Supplement: Supplementary Information [file srep45759-s1.pdf]

Supplementary Material for 'Enhanced methane emissions from tropical wetlands during the 2011 La Niña'

Sudhanshu Pandey[1,2,*], Sander Houweling[1,2], Maarten Krol[1,2,3], Ilse Aben[2], Guillaume Monteil[4], Narcisa Nechita-Banda[2], Edward J. Dlugokencky[5], Rob Detmers[2], Otto Hasekamp[2], Xiyan Xu[6], William J. Riley[6], B. Poulter[7], Z. Zhang[8], K. C. McDonald[9], James W. C. White[10], Philippe Bousquet[11], and Thomas Röckmann[1]

[1] Institute of Marine and Atmospheric Research Utrecht (IMAU), Utrecht University, The Netherlands

[2]SRON Netherlands institute for Space Research, Utrecht, The Netherlands

[3]Department of Meteorology and Air Quality (MAQ), Wageningen University and Research Centre, Wageningen

[4]Department of Physical Geography and Ecosystem Science, Lund University, Lund, Sweden

[5]NOAA Earth System Research Laboratory, Boulder, Colorado, USA

[6]Earth Sciences Division, Lawrence Berkeley National Laboratory, Berkeley, California, USA

[7]Institute on Ecosystems and Department of Ecology, Montana State University, Bozeman, USA

[8]Swiss Federal Research Institute WSL, Dynamics Macroecology, Zürcherstrasse 111, Birmensdorf 8903, Switzerland

[9]City College of New York, City University of New York, New York, NY, USA

[10]Institute of Arctic and Alpine Research, Boulder, CO, USA

[11]Laboratoire des Sciences du Climatet de l'Environnement, CEA-CNRS-UVSQ, F-91191, France

[*]corresponding author (s.pandey@uu.nl)

January 30, 2017

1 TM5

The global atmospheric tracer transport model TM5 [*Krol et al.*, 2005] simulates the spatiotemporal distribution of a tracer in the atmosphere, for a given set of surface emissions

and atmospheric sinks. We run it at 6° × 4° (longitude × latitude) horizontal resolution and 25 vertical hybrid sigma pressure levels from the surface to the top of the atmosphere. The meteorological fields for this offline model are taken from the European Centre for Medium-Range Weather Forecasts (ECMWF) ERA-Interim reanalysis [*Dee et al.*, 2011]. Our version of the model uses ERA-Interim archived convective mass fluxes.

In a TM5-Meteorology simulation, an initial spin up of 12 years was to bring the atmospheric mole fractions and emissions of CH_4 in equilibrium. Tropospheric oxidation of methane is calculated using the monthly distribution of OH from *Spivakovsky et al.* [2000], adjusted by a single scaling factor of 0.92, derived from inverse modeling of methyl chloroform in TM5. The same seasonally varying OH field is repeated every year. The modeled vertical profiles were sampled at locations of GOSAT soundings and converted into total columns using GOSAT averaging kernels [*Monteil et al.*, 2013].

The meteorology simulation of the $\delta^{13}C\text{-}CH_4$ version of TM5 [*Monteil et al.*, 2011] was given a spin up of 30 years to bring atmospheric $\delta^{13}C\text{-}CH_4$ in the model in equilibrium with $\delta^{13}C\text{-}CH_4$ signature of emissions.

2 Inverse modeling

The TM5-4DVAR inverse modeling system was used to estimate surface CH_4 emissions measurements of the dry air mole fraction of methane in flask samples from the surface network as well as satellite retrievals of its column average mole fraction. It comprises of TM5 coupled to a variational data assimilation system (4DVAR, *Meirink et al.* [2008]). CH_4 emissions are optimized as a single category representing the sum of all source and sink processes, for each surface grid box of TM5 and each month of simulation. For anthropogenic emissions, we use the 4.2FT2010 version of EDGAR (European Commission, Joint Research Centre (JRC)/Netherlands Environmental Assessment Agency). *A priori* emissions from other processes were used as described in *Houweling et al.* [2014]. The covariance matrix of the state vector of surface emissions is constructed assuming a relative emission uncertainty of 50% per grid box per month for the 'total' CH_4 category. The emissions are assumed to be correlated temporally using an exponential function with a correlation length of 3 months, and spatially using a Gaussian function with a decorrelation length scale of 500 km.

3 Uncertainty estimation

In Figures 1 and 2 of the main text, the shaded regions represent $\pm 1\sigma$ uncertainty of the respective time series. These uncertainties were calculated using the Monte-Carlo method with 100 simulations. In Figure 1, the retrievals errors, provided by RemoteC, were assigned as uncertainty of individual GOSAT XCH_4 measurements. For model output, model representation errors were used. In Figure 2, the emission uncertainties are the posterior uncertainties calculated by TM5-4DVAR [*Basu et al.*, 2013]. $\delta^{13}C\text{-}CH_4$ measurements were assigned an uncertainty of 0.02 ‰. Similar procedures were used in SM Figure 1 and 5.

4 Surface measurements of CH_4

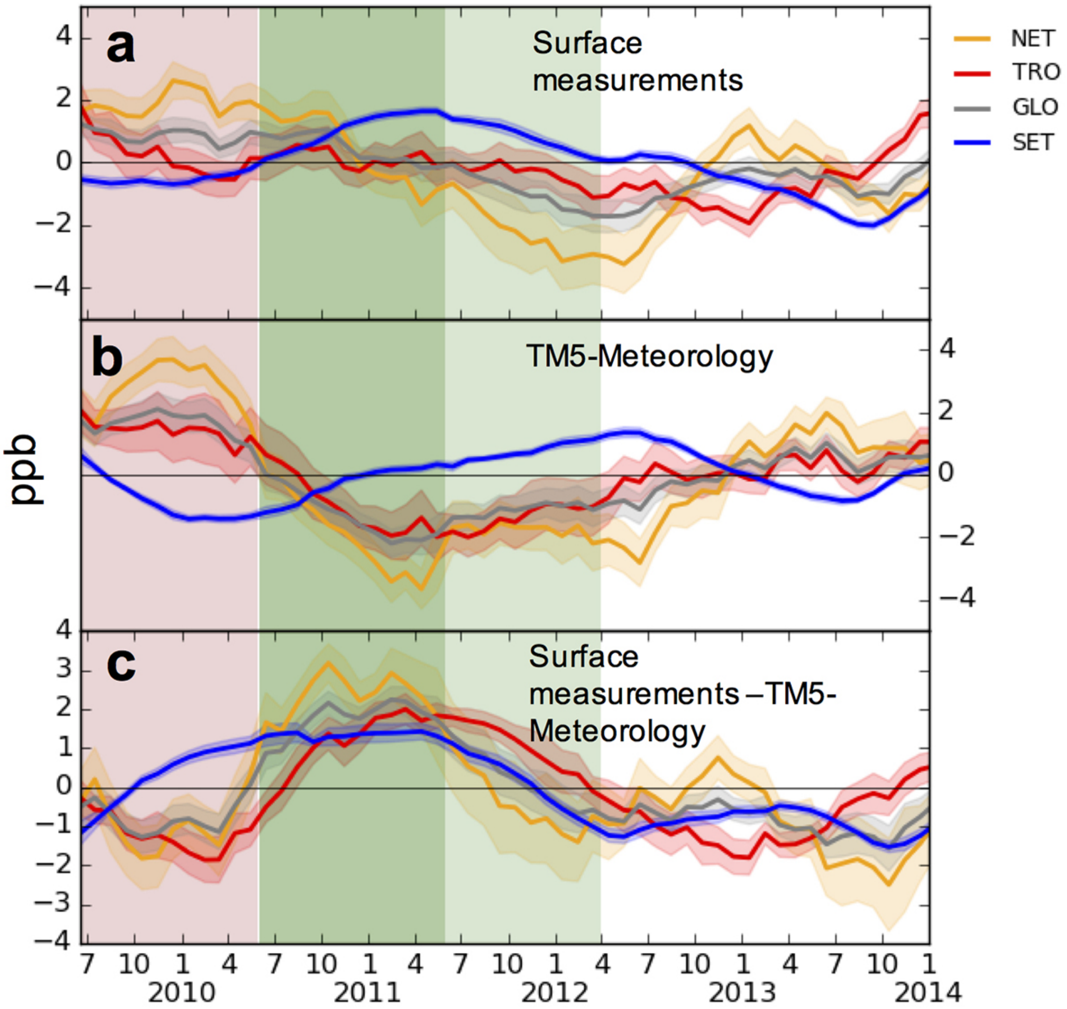

Figure 1: Same as Figure 1 in the main text, but for surface flask-air measurements of CH_4 from the NOAA and CSIRO networks.

Surface measurements of CH_4 are shown in Figure 1. There are considerable differences between the zonal mean CH_4 variations derived from these data and the GOSAT FP retrievals (see Figure 1 of main text). Unlike GOSAT FP ($\Delta_{\mathrm{TRO}}^{XCH_4}$= 2.42 ppb), there is no enhancement in TRO in surface data ($\Delta_{\mathrm{TRO}}^{CH_4} = -0.29$ ppb) during LN11. Enhancement in SET is also lower (surface $\Delta_{\mathrm{SET}}^{CH_4} = 1.52$ ppb vs. GOSAT FP $\Delta_{\mathrm{SET}}^{XCH_4} = 5.62$ ppb). Also, there is a significant decrease in TRO due to meteorology ($\Delta_{\mathrm{TRO}}^{CH_4} = -2.62$ ppb) during LN11,

which is not seen in GOSAT FP.

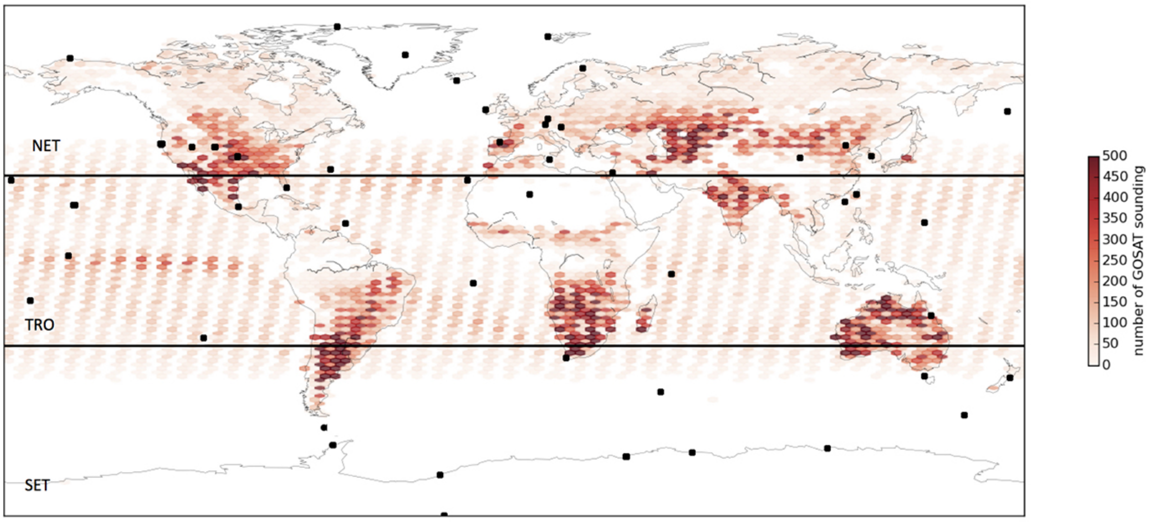

Figure 2: Coverage of GOSAT RemoteC full-physics XCH_4 and the NOAA and CSIRO surface air sampling sites (black dots) that were used. Only surface sites with continuous coverage between 2009-2015 and at least monthly temporal resolution were used. This map was generated using python v2.7 with matplotlib-basemap library [*Hunter*, 2007]

The differences in between the interannual variation in surface measurements and satellite retrievals likely reflect differences in spatial coverage of the two datasets. GOSAT FP has a more even spatial coverage than the surface networks (see Figure 2). A larger number of tropical surface measurements are taken in the northern hemisphere compared with southern hemisphere, which can bias the zonal average. Also, the signal from land takes quite a long transport path—including upward transport by convection, etc.—before reaching a marine site in the Tropics, increasing the chance of transport variations modifying the signal.

After subtracting the TM5-Meteorology, influences of sampling and transport are removed from the two measurement sets and their residual anomalies are in better agreement (surface $\Delta_{\mathrm{TRO}}^{CH_4} = 2.34$ ppb vs. GOSAT $\Delta_{\mathrm{TRO}}^{XCH_4} = 2.04$ppb). This is consistent with the posterior emissions from the inversion assimilating only surface measurements being similar to those obtained when assimilating GOSAT FP and surface measurements (see Figure 3).

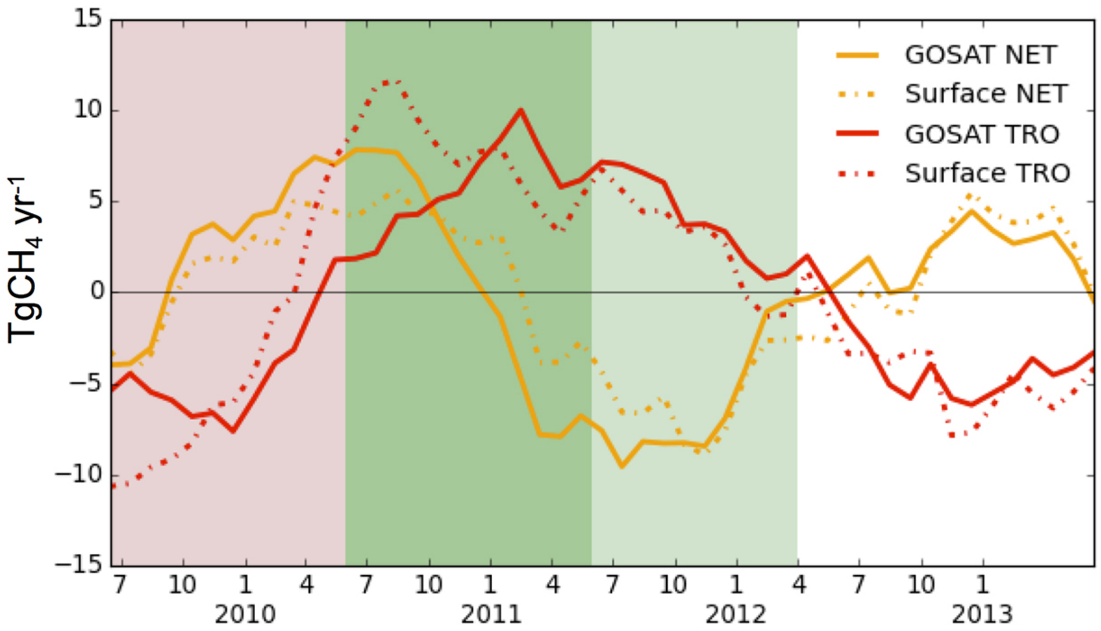

Figure 3: Detrended and smoothened TM5-4DVAR CH_4 emissions estimated after assimilating GOSAT FP and/or surface data.

5 Biomass burning emissions

We use the GFED4s inventory to account for CH_4 emission from biomass burning (BB). GFED uses remotely sensed fire activity and vegetation productivity to derive gridded monthly burned area and resulting BB emissions [*Van Der Werf et al.*, 2010]. The variability of BB emissions is shown in Figure 4. It do not suggest that biomass burning contributed to the increased emissions during the La Nina. It is smaller ($1\sigma = \pm 2$ $TgCH_4\,yr^{-1}$) than the variability of optimized total CH_4 emissions (see SM figure 4). *Bousquet et al.* [2006] found that BB-related variations generally contributes 15% to the total emission anomalies. High BB emissions are observed in mid-2012 after the La Niña. This might result from the building up of biomass fuel during the preceding La Niña phases with anomalously wet condition in regions like Australia [*Detmers et al.*, 2015].

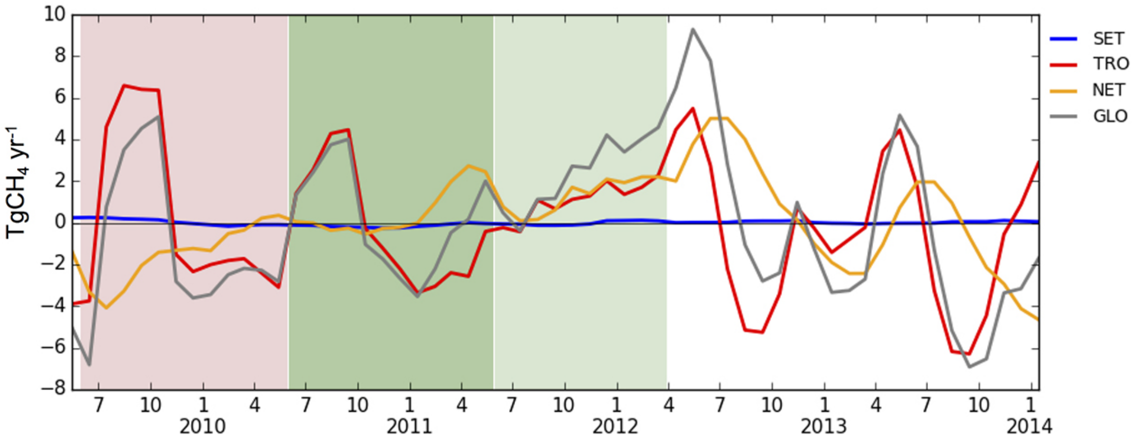

Figure 4: Detrended and smoothened biomass burning CH_4 emissions derived from GFED4s.

6 Interannual variability of the CH_4 sink

To investigate the possible influence of variations in the OH sink on CH_4, we compare the optimized CH_4 emissions from LMDz-PYVAR-SACS [*Locatelli et al.*, 2015; *Chevallier et al.*, 2005; *Hourdin et al.*, 2006] inversion with TM5-4DVAR inversion (See Figure 5). The prior emissions used in these inversions do not account for any interannual variability. LMDz-PYVAR-SACS has annually repeating prior emissions for all categories. The prior emissions of TM5-4DVAR also have no inter annual variability except for biomass burning emissions that are taken from GFED4s. This does not affect our final analysis as GFED4s CH_4 emissions are subtracted from the posterior beforehand.

In LMDz-PYVAR-SACS, OH fields were optimized using methyl chloroform (MCF) measurements. These results should be treated with caution as

1. The MCF-based OH optimization becomes increasingly uncertain with MCF levels dropping to only a few ppt in recent years.

2. It is difficult to determine the correct relative uncertainties of CH_4 and MCF, which introduces a temporally varying weight of the MCF measurements on the solution of the coupled inversion system.

3. We make a comparison between different inversion systems. Doing so complicates the comparison, especially for the absolute optimized emissions as different inversion

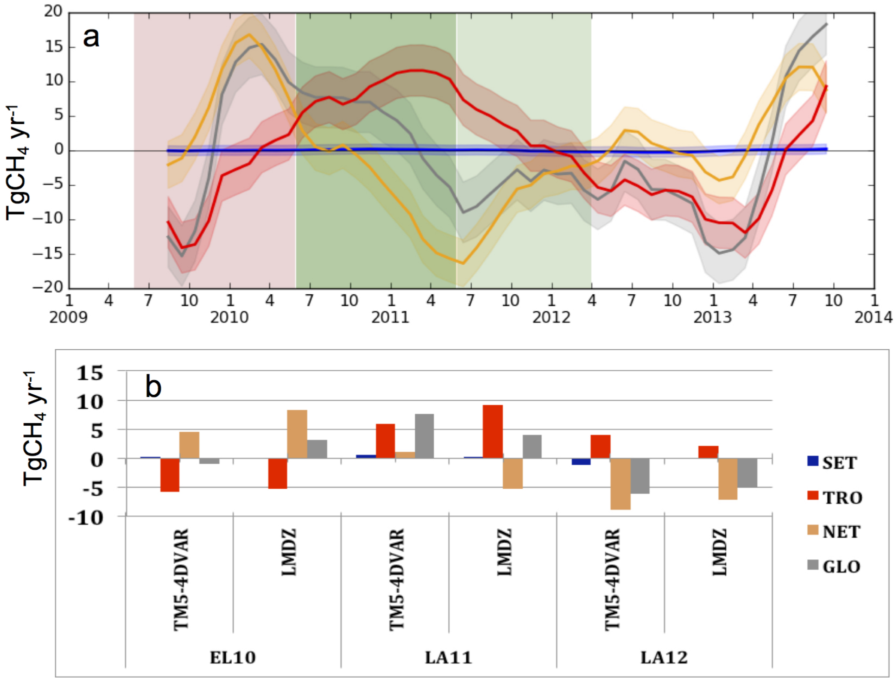

Figure 5: Results of LMDz-PYVAR-SACS inversion. a) Detrended and smoothened posterior CH_4 emissions with $\pm 1\sigma$ uncertanities. b) Comparison of the LMDz-PYVAR-SACS and TM5-4DVAR derived μ^{emission} (mean during the three ENSO phases).

systems may give a wide range of estimates depending on their setup and boundary conditions. However, inversion-optimized temporal emission variations are known to be less sensitive to differences in inversion setup than the mean state.

In addition, recent studies have pointed out that atmospheric OH is well buffered against changes in its driving parameters [*Lelieveld et al.*, 2016]. The δ^{13}C-CH_4 influence of a 3 $TgCH_4\,yr^{-1}$ enhanced sink will only be 0.005 ‰, which is within the error margins of the δ^{13}C-CH_4 anomalies. If the whole anomaly was caused by OH this would lead to an isotopic effect that was less than observed, suggesting the observed anomaly is driven by changes in the sources rather than the sinks.

7 Transport impact on δ^{13}C-CH_4

Figure 2 in the main text shows the XCH_4 variability due to variability in atmospheric transport. Meteorological variability can influence δ^{13}C-CH_4 due to the isotopic fractiona-

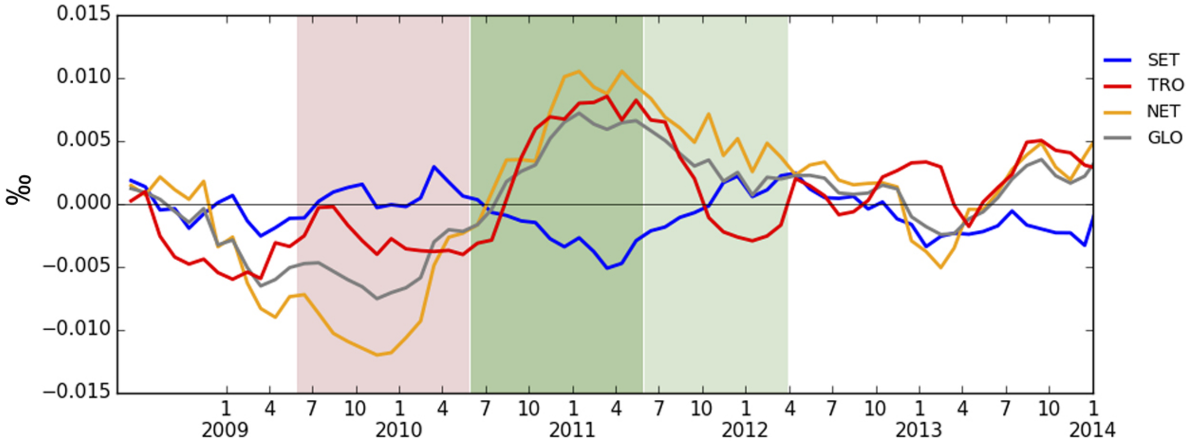

Figure 6: Detrended and smoothened δ^{13}C-CH_4 at NOAA air sampling sites from a TM5-Meteorology simulation.

tion of the OH sink, changes in the strength of inter-hemispheric exchange, etc. Figure 6 shows the simulated δ^{13}C-CH_4 variability in response to variations in transport. Overall, they are an order of magnitude less than the variability in the δ^{13}C-CH_4 measurements.

8 Process-based wetland models

Process-based wetland models estimate CH_4 emissions from natural wetlands using information about precipitation, temperature, biomass availability, etc. We analyzed the CH_4 emission output from two such models: LPJ-wsl [*Hodson et al.*, 2011; *Zhang et al.*, 2016] and CLM4.5 (referred as CLM from here on) [*Riley et al.*, 2011; *Xu et al.*, 2016]. These models show a weaker enhancement of CH_4 emissions during LN11 than the TM5-4DVAR inversion (See Figure 7). A poor correlation is seen between these emissions and precipitation anomalies. This happens despite general agreement between the inundated area calculated by the hydrological schemes of these bottom-up models and SWAMPS (Surface WAter Microwave Product Series). As shown in the main text (see Figure 3) the inundated area in SWAMPS correlates well with the inversion derived emission anomalies in TRO.

Two mechanisms, that are implemented in the process-based wetland models, might explain the disagreement between inundated area and modeled CH_4 emissions:

1. CH_4 emission is directly related to ecosystem respiration, which increases with increasing temperature. During LA11 the temperature anomaly in TRO was slightly negative ($\mu_{\text{TRO}}^{\text{temperature}} = -0.05°\text{C}$), and hence, it will decrease the strength of inundation-driven positive CH_4 emission anomaly.

2. The relation between extent of inundated area and CH_4 emission is complex. In general, wetland CH_4 emission increases with increase in inundated area. However, the reverse can also happen if the increase in precipitation causes a higher water table depth, which will increase the chances of CH_4 getting oxidized before reaching the atmosphere.

Higher CH_4 emissions are observed during LN11 in CLM ($\mu_{\text{TRO}}^{\text{emission}} = 2.38\ \text{TgCH}_4\,\text{yr}^{-1}$) than in LPJ-wsl ($\mu_{\text{TRO}}^{\text{emission}} = 1.54\ \text{TgCH}_4\,\text{yr}^{-1}$). This might be as CLM ($\approx 250\ \text{TgCH}_4\,\text{yr}^{-1}$) has a higher annual global emission than LPJ-wsl ($\approx 170\ \text{TgCH}_4\,\text{yr}^{-1}$). *Bohn et al.* [2015] highlighted the large uncertainties in present wetland models. They could be analyzed in further detail using our inversion estimates.

9 Other retrieval/inversion methods

An important source of systematic error in satellite retrievals is the scattering of light by aerosols and thin cirrus clouds along the measured light path. The full-physics (FP, *Butz et al.* [2010]) and the proxy [*Frankenberg et al.*, 2005] retrieval methods have been developed in the past to account for such atmospheric scattering. Additionally, the so-called *ratio* method assimilates X_{ratio} (XCH_4:XCO_2) directly to optimize the surface fluxes of CH_4 and CO_2 [*Fraser et al.*, 2014; *Pandey et al.*, 2015, 2016]. Hence, it avoids the errors introduced in translating retrieved X_{ratio} to XCH_4 using modeled XCO_2 (XCO_2^{model}).

The proxy and *ratio* method generally yield twice as many valid CH_4 retrievals as FP, because the latter requires stricter cloud filtering criteria. In this study, we still use FP retrievals to avoid potential correlations between the inter-annual variations of CH_4 and CO_2 in response to ENSO. The proxy retrieval method might erroneously attribute an CO_2 anomaly, that is not captured in XCO_2^{model}, to XCH_4. Figure 8 illustrates that this is indeed what happens. The *ratio* inversion method does not depend on the XCO_2^{model}, however, it can still wrongly assign a CO_2 anomaly to CH_4 emissions to fit the X_{ratio} in the atmosphere

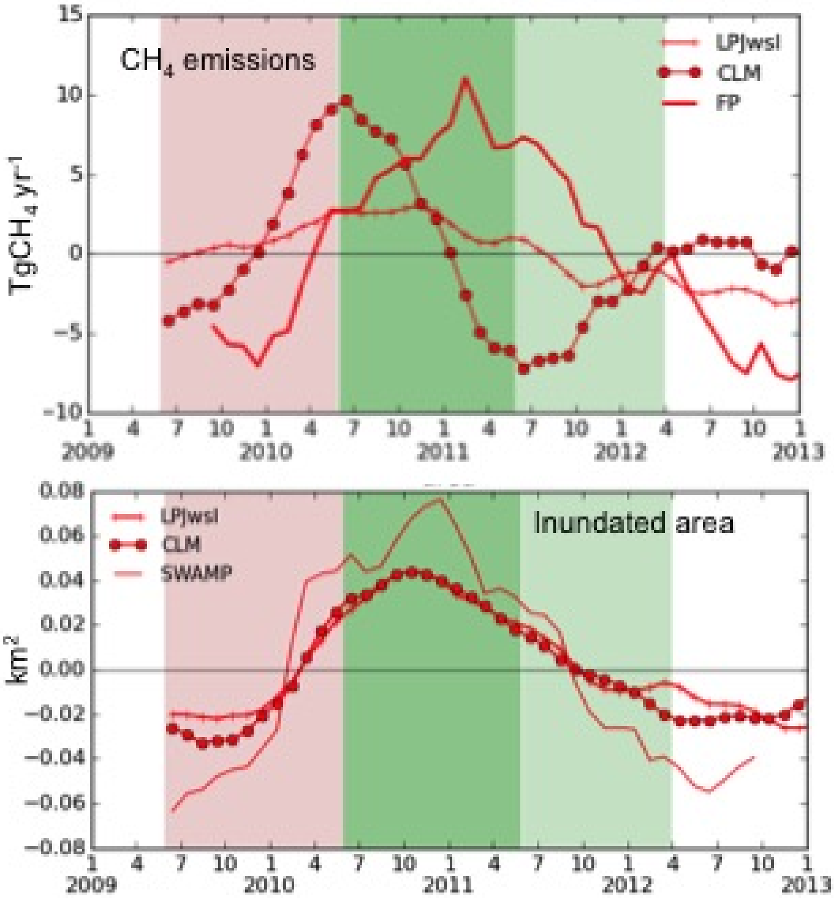

Figure 7: Detrended and smoothened CH_4 emissions and total inundated area in the Tropics estimated by wetland models and SWAMPS.

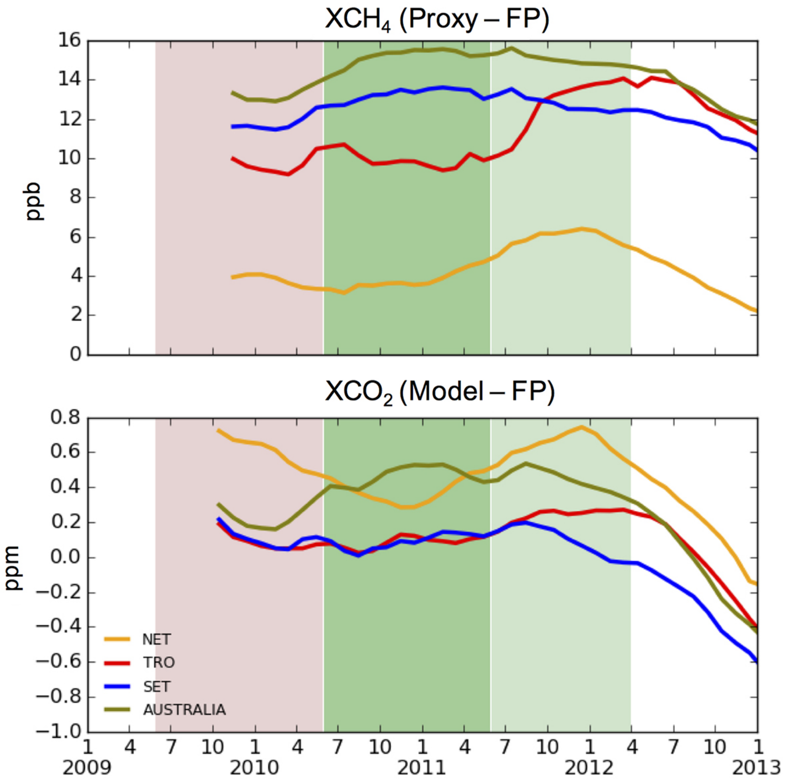

Figure 8: The impact of XCO_2^{model} on CH_4 proxy retrievals. There is high correlation (R) between proxy − FP XCH_4, and XCO_2^{model} − FP XCO_2 (R values: TRO = 0.41, NET = 0.90, SET = 0.92, Australia = 0.97) indicating that an important fraction of the variability in proxy XCH_4 might be wrongly attributed CO_2 variability.

depending on the relative uncertainty assigned to the *a priori* CO_2 and CH_4 fluxes.

Figure 9 shows CH_4 emissions derived for TRO with the different inversion methods. Overall, the variabilities of the emissions are in agreement. During LN11, the *ratio* ($\mu_{\text{TRO}}^{\text{emission}}$ =7.03 $\text{TgCH}_4\,\text{yr}^{-1}$) and proxy ($\mu_{\text{TRO}}^{\text{emission}}$ =9.54 $\text{TgCH}_4\,\text{yr}^{-1}$) methods estimate a larger positive anomaly than FP inversion. During LN11, there was a negative CO_2 anomaly globally, driven by increased vegetation growth in semi-arid regions of the Southern Hemisphere notably Australia [*Detmers et al.*, 2015]. XCO_2^{model} and the *ratio* method optimized CO_2 emissions do not properly account for this and hence, cause an incorrect attribution to CH_4. Therefore, the *ratio* and proxy methods find larger anomalies than the FP inversion. The opposite is seen during EN10 and after LN12.

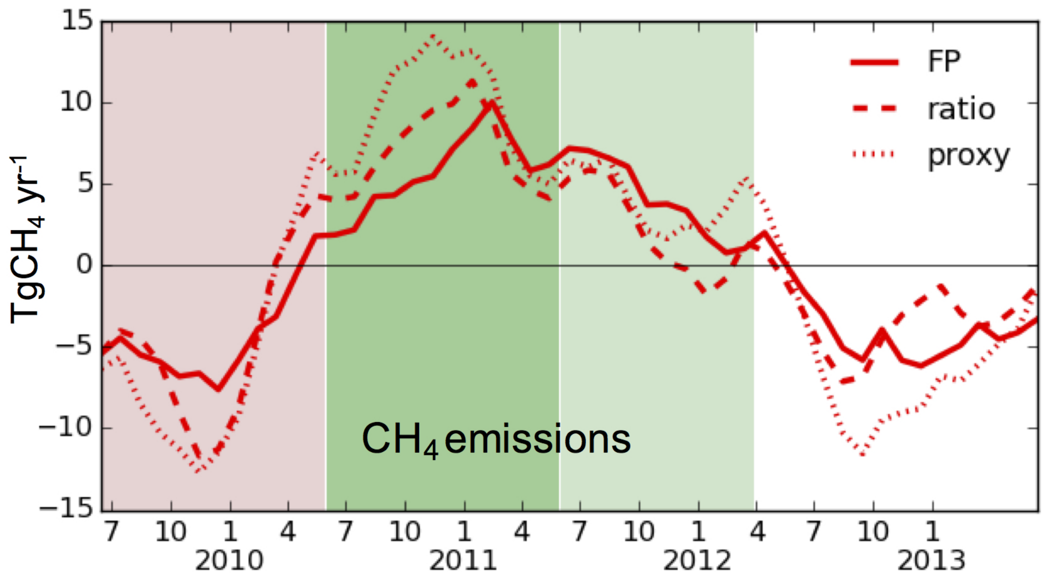

Figure 9: Detrended and smoothened CH_4 emission from the *ratio* inversion and inversions assimilating proxy and FP CH_4 retrievals. All these inversions also assimilate surface observations.

9.1 Climate Variations in the Tropics

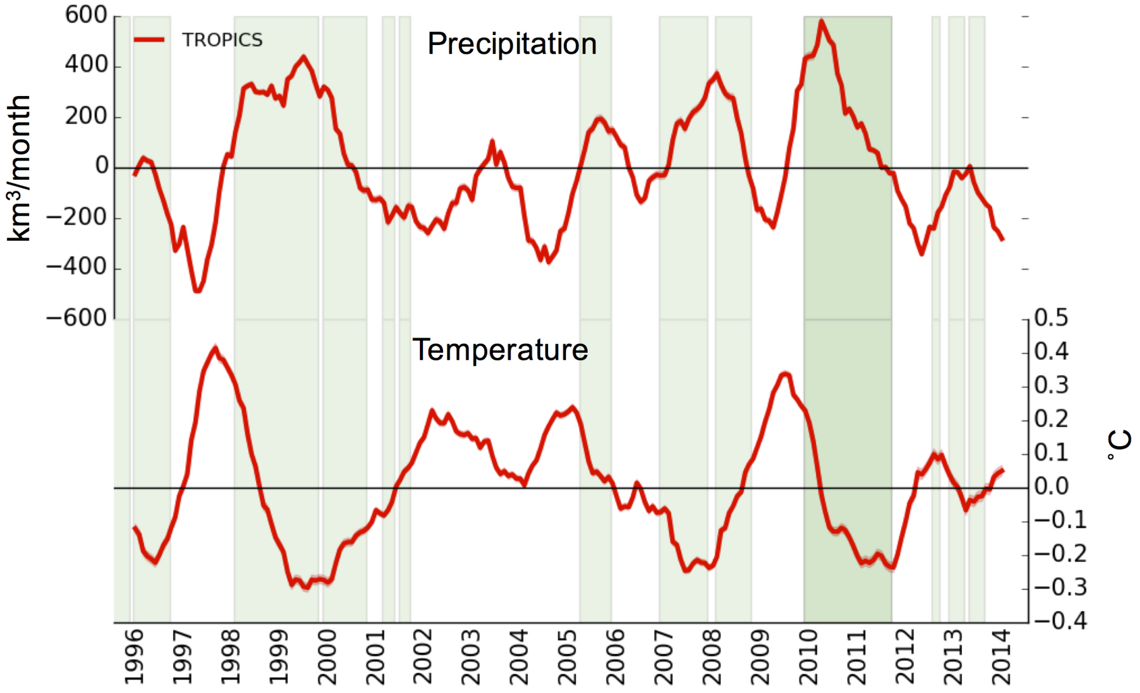

Figure 10: Detrended and smoothened monthly precipitation and temperature times series of the Tropics from CRU-TS v3.23. The vertical green spans represent La Niña episodes.

9.2 Correlation analysis

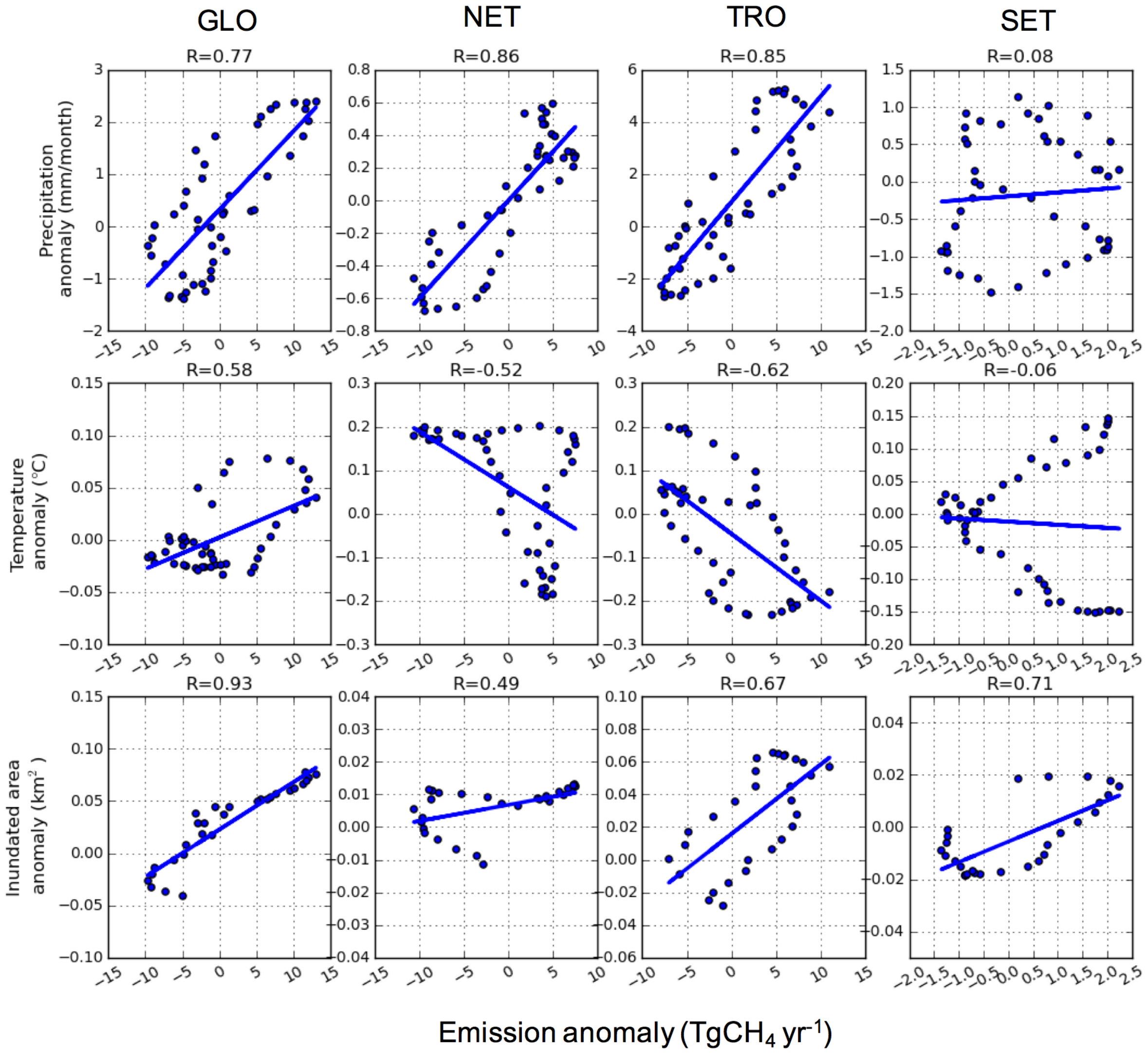

Figure 11: Pearson product-moment correlation (R) between monthly anomalies of TM5-4DVAR CH_4 emissions, derived by assimilating FP XCH_4, and anomalies of temperature, precipitation and inundated area.

References

Basu, S., et al., Global CO_2 fluxes estimated from GOSAT retrievals of total column CO_2, *Atmospheric Chemistry and Physics*, *13*(17), 8695–8717, doi:10.5194/acp-13-8695-2013, 2013.

Bohn, T. J., et al., WETCHIMP-WSL: intercomparison of wetland methane emissions models over West Siberia, *Biogeosciences*, *12*, 3321–3349, doi:10.5194/bg-12-3321-2015, 2015.

Bousquet, P., et al., Contribution of anthropogenic and natural sources to atmospheric methane variability., *Nature*, *443*(7110), 439–43, doi:10.1038/nature05132, 2006.

Butz, A., O. P. Hasekamp, C. Frankenberg, J. Vidot, and I. Aben, CH_4 retrievals from space-based solar backscatter measurements: Performance evaluation against simulated aerosol and cirrus loaded scenes, *Journal of Geophysical Research*, *115*(D24), D24,302, doi:10.1029/2010JD014514, 2010.

Chevallier, F., M. Fisher, P. Peylin, S. Serrar, P. Bousquet, F. M. Bréon, A. Chédin, and P. Ciais, Inferring CO_2 sources and sinks from satellite observations: Method and application to TOVS data, *Journal of Geophysical Research: Atmospheres*, *110*(24), 1–13, doi:10.1029/2005JD006390, 2005.

Dee, D. P., et al., The ERA-Interim reanalysis: configuration and performance of the data assimilation system, *Quarterly Journal of the Royal Meteorological Society*, *137*(656), 553–597, doi:10.1002/qj.828, 2011.

Detmers, R. G., et al., Anomalous carbon uptake in Australia as seen by GOSAT, *Geophysical Research Letters*, *42*(19), 8177–8184, doi:10.1002/2015GL065161, 2015.

Frankenberg, C., J. F. Meirink, M. van Weele, U. Platt, and T. Wagner, Assessing methane emissions from global space-borne observations., *Science (New York, N.Y.)*, *308*(5724), 1010–4, doi:10.1126/science.1106644, 2005.

Fraser, A., P. I. Palmer, L. Feng, H. Bösch, R. Parker, E. J. Dlugokencky, P. B. Krummel, and R. L. Langenfelds, Estimating regional fluxes of CO_2 and CH_4 using space-borne observations of XCH_4: XCO_2, *Atmospheric Chemistry and Physics*, *14*(23), 12,883–12,895, doi:10.5194/acp-14-12883-2014, 2014.

Hodson, E. L., B. Poulter, N. E. Zimmermann, C. Prigent, and J. O. Kaplan, The El Nio-Southern Oscillation and wetland methane interannual variability, *Geophysical Research Letters*, *38*(8), 3–6, doi:10.1029/2011GL046861, 2011.

Hourdin, F., et al., The LMDZ4 general circulation model: climate performance and sensitivity to parametrized physics with emphasis on tropical convection, *Climate Dynamics*, *27*(7), 787–813, doi:10.1007/s00382-006-0158-0, 2006.

Houweling, S., et al., A multi-year methane inversion using SCIAMACHY, accounting for systematic errors using TCCON measurements, *Atmospheric Chemistry and Physics*, *14*(8), 3991–4012, doi:10.5194/acp-14-3991-2014, 2014.

Hunter, J. D., Matplotlib: A 2d graphics environment, *Computing In Science & Engineering*, *9*(3), 90–95, doi:10.1109/MCSE.2007.55, 2007.

Krol, M., S. Houweling, B. Bregman, M. van den Broek, A. Segers, P. van Velthoven, W. Peters, F. Dentener, and P. Bergamaschi, The two-way nested global chemistry-transport zoom model TM5: algorithm and applications, *Atmospheric Chemistry and Physics*, *5*(2), 417–432, doi:10.5194/acp-5-417-2005, 2005.

Lelieveld, J., S. Gromov, A. Pozzer, and D. Taraborrelli, Global tropospheric hydroxyl distribution, budget and reactivity, *Atmospheric Chemistry and Physics Discussions*, pp. 1–25, doi:10.5194/acp-2016-160, 2016.

Locatelli, R., P. Bousquet, M. Saunois, F. Chevallier, and C. Cressot, Sensitivity of the recent methane budget to LMDz sub-grid-scale physical parameterizations, *Atmospheric Chemistry and Physics*, *15*, 9765–9780, doi:10.5194/acp-15-9765-2015, 2015.

Meirink, J. F., P. Bergamaschi, and M. C. Krol, Four-dimensional variational data assimilation for inverse modelling of atmospheric methane emissions: method and comparison with synthesis inversion, *Atmospheric Chemistry and Physics Discussions*, *8*(3), 12,023–12,052, doi:10.5194/acpd-8-12023-2008, 2008.

Monteil, G., S. Houweling, E. J. Dlugockenky, G. Maenhout, B. H. Vaughn, J. W. C. White, and T. Röckmann, Interpreting methane variations in the past two decades using measurements of CH_4 mixing ratio and isotopic composition, *Atmospheric Chemistry and Physics*, *11*(17), 9141–9153, doi:10.5194/acp-11-9141-2011, 2011.

Monteil, G., et al., Comparison of CH_4 inversions based on 15 months of GOSAT and SCIAMACHY observations, *Journal of Geophysical Research: Atmospheres*, *118*, 11,807–11,823, doi:10.1002/2013JD019760, 2013.

Pandey, S., S. Houweling, M. Krol, I. Aben, and T. Röckmann, On the use of satellite-derived CH_4 : CO_2 columns in a joint inversion of CH_4 and CO_2 fluxes, *Atmospheric Chemistry and Physics*, *15*, 8615–8629, doi:10.5194/acp-15-8615-2015, 2015.

Pandey, S., et al., Inverse modeling of GOSAT-retrieved ratios of total column CH_4 and CO_2 for 2009 and 2010, *Atmospheric Chemistry and Physics*, *16*(8), 5043–5062, doi: 10.5194/acp-16-5043-2016, 2016.

Riley, W. J., Z. M. Subin, D. M. Lawrence, S. C. Swenson, M. S. Torn, L. Meng, N. M. Mahowald, and P. Hess, Barriers to predicting changes in global terrestrial methane fluxes: Analyses using CLM4Me, a methane biogeochemistry model integrated in CESM, *Biogeosciences*, *8*(7), 1925–1953, doi:10.5194/bg-8-1925-2011, 2011.

Spivakovsky, C. M., et al., Three-dimensional climatological distribution of tropospheric OH: Update and evaluation, *Journal Of Geophysical Research-Atmospheres*, *105*(D), 8931–8980, doi:10.1029/1999JD901006, 2000.

Van Der Werf, G. R., et al., Global fire emissions and the contribution of deforestation, savanna, forest, agricultural, and peat fires (1997-2009), *Atmospheric Chemistry and Physics*, *10*(23), 11,707–11,735, doi:10.5194/acp-10-11707-2010, 2010.

Xu, X., et al., A multi-scale comparison of modeled and observed seasonal methane cycles in northern wetlands, *Biogeosciences*, *13*, 5043–5056, doi:10.5194/bg-2016-167, 2016.

Zhang, Z., N. E. Zimmermann, J. O. Kaplan, and B. Poulter, Modeling spatiotemporal dynamics of global wetlands: Comprehensive evaluation of a new sub-grid TOPMODEL parameterization and uncertainties, *Biogeosciences*, *13*(5), 1387–1408, doi: 10.5194/bg-13-1387-2016, 2016.
